# A New Type of Composite Membrane PVA-NaY/PA-6 for Separation of Industrially Valuable Mixture Ethanol/Ethyl *Tert*-Butyl Ether by Pervaporation

**DOI:** 10.3390/ma13173676

**Published:** 2020-08-20

**Authors:** Katarzyna Knozowska, Joanna Kujawa, Renars Lagzdins, Alberto Figoli, Wojciech Kujawski

**Affiliations:** 1 Faculty of Chemistry, Nicolaus Copernicus University in Toruń, 7 Gagarina Street, 87-100 Toruń, Poland; katkno@doktorant.umk.pl (K.K.); joanna.kujawa@umk.pl (J.K.); renars.lagzdins1@gmail.com (R.L.); 2 Faculty of Nature Sciences and Mathematics, Daugavpils University, 1 Parādes Street, LV-5401 Daugavpils, Latvia; 3 Institute on Membrane Technology, CNR-ITM, Via P. Bucci 17c, 87030 Rende, Italy; a.figoli@itm.cnr.it; 4 National Research Nuclear University MEPhI, 31 Kashirskoe Hwy, Moscow 115409, Russia

**Keywords:** preparation and characterization of composite membranes, NaY zeolite, poly(vinyl alcohol), poly(amide-6), organic–organic pervaporation, ethanol/ethyl *tert*-butyl ether (EtOH/ETBE) mixture

## Abstract

Pervaporation is a membrane technique used to separate azeotropic and close boiling solvents. Heterogenous PVA composite membranes with NaY zeolite supported on polyamide-6 were fabricated and utilized in organic–organic pervaporation. The efficiency of prepared membranes was evaluated in the separation of ethanol/ethyl *tert*-butyl ether (EtOH/ETBE) using separation factor (*β*) and the thickness normalized pervaporation separation index (*PSI_N_*). Implementation of the fringe projection phase-shifting method allowed to the determined contact angle corrected by roughness. The influence of the presence of water traces in the feed on the overall separation efficiency was also discussed using the enrichment factor for water (*EF_water_*). The incorporation of NaY into PVA matrix increases surface roughness and hydrophilicity of the composite membrane. It was found that membranes selectively transport ethanol from the binary EtOH/ETBE mixture. The values of *β* (2.3) and *PSI_N_* (288 μm g m^−2^ h^−1^) for PVA-NaY/PA6 membrane were improved by 143% and 160% in comparison to the values for the pristine PVA/PA6 membrane. It was found that membranes showed *EF_water_* > 1, thus revealing the preferential transport of water molecules across membranes. These results are also significant for the design of membranes for the removal of water excess from the mixtures of organic solvents.

## 1. Introduction

Nowadays, environmental pollutions have become one of the major problems with which governments and societies are facing. Therefore, the sustainable production of fuels characterized by a lower environmental impact is an approach for reducing pollutant emissions. Moreover, the new, more strict law has been implemented to force refineries to produce gasoline without addition of lead and to reduce the content of aromatic compounds [1].

In the 1990s, methyl *tert*-butyl ether (MTBE) was commonly used as an octane enhancer in the United States of America. However, the biodegradation of MTBE is very difficult, and MTBE possesses toxic properties. The presence of MTBE in groundwater and drinking water was detected around the USA, especially in urban areas [2,3]. This presence was caused by the leakage from the underground storage tanks of MTBE [2,3]. Taking into account the toxicity of MTBE, the government of the USA decided to ban the addition of MTBE in gasoline [2]. Ethyl tert-butyl ether (ETBE)could be the replacement of the toxic MTBE [4,5]. ETBE possesses lower volatility and water solubility, better antiknock properties in comparison to MTBE. On the industrial scale, ETBE is produced by the reaction of isobutene with an excess of ethanol (EtOH) in the presence of an ion-exchange resin catalyst at below 80 °C and at the pressure of 0.6 MPa [4,6]. The final mixture consists of unreacted EtOH and ETBE, which form the azeotropic mixture containing 20 wt % of ethanol and 80 wt % of ethyl *tert*-butyl ether [4,6]. Separation azeotropic mixtures require the addition of the third component (e.g., water) [7]. It should be also mentioned that ethanol used in the ETBE synthesis can be obtained from renewable sources [3]. Ethyl tert-butyl ether is an industrially crucial organic solvent. Therefore, it is necessary to find separation techniques lowering energy consumption. Membrane separation processes can be a real alternative to the ternary distillation process [7].

One of the membrane separation technique used for the separation of liquid mixtures is pervaporation [8,9]. Due to its characteristics, pervaporation shows lower energy consumption than the traditional separation methods and can be successfully implemented for the separation of azeotropic and close boiling mixtures [10,11,12,13]. 

Separation of EtOH/ETBE mixtures have been investigated recently focusing on the application on various hydrophilic polymers such as poly(lactide), poly(vinyl pyrrolidone), poly(amide-imide), poly(urethane-amide-imide), and cellulose acetate [1,7,14,15,16,17,18]. Furthermore, researchers developed polymeric membranes filled with inorganic additives to improve the overall membrane performance and material features. Zhu et al. [19] described the application of NaY zeolite membrane for the EtOH/ETBE separation. Prepared membrane showed good long term stability, stable total flux, and excellent separation ability [19]. 

Zeolites are porous crystalline framework materials consisting of the SiO_4_ and AlO_4_ tetrahedrons [20]. These tetrahedrons are connected by sharing the atom of oxygen. Aluminum in the center of AlO_4_ tetrahedron is negatively charged and can be compensated by exchangeable metal cation (Na^+^, K^+^) [20]. NaY is a part of the faujasite (FAU) group of the zeolites [21]. NaY is characterized by a large pore size equal to 7.4Å and a size cage equal to 11.8Å [22]. Moreover, this zeolite possesses a durable hydrophilic nature and high resistance to water. Due to the excellent properties, NaY has been chosen as a membrane material for the dehydration of alcohol [23]. FAU type membranes were also applied for ethanol dehydration and separation of organic solvent mixtures (alcohol/benzene, cyclohexane, methyl *tert*-butyl ether, or ethyl *tert*-butyl ether) by pervaporation [24]. Zeolite NaY membranes were prepared by the continuous intergrowth of zeolite crystals on a porous substrate. Pervaporation experiments showed that NaY membrane selectively transports water from the water–ethanol mixtures (*β_H2O/EtOH_* = 170 (10% H_2_O). In the case of mixtures of organic solvent, it was noticed that the membrane was selective toward ethanol and methanol (mixtures of ethanol/benzene, methanol/benzene) while during the separation of benzene/*n*-hexane and benzene/cyclohexane mixtures, membrane selectively transports benzene from the mixtures [24]. 

Rhim and Kim [25] investigated the efficiency of crosslinked, blended PVA/PAA membranes in the separation of methanol from methyl *tert*-butyl ether/methanol mixture. Pervaporation results showed that PVA/PAA (85/15) membrane exhibited very good separation properties (*β_MeOH/MTBE_* = 4000 (20% MeOH)). Despite the excellent separation ability, this membrane was characterized by a low value of total flux (*J_t_* = 10.1 g m^−2^ h^−1^) [25]. 

The preparation of composite membranes can overcome the problem of very low transport properties of membranes. These types of membranes consist of a thin dense selective layer and porous support. The idea of this solution is to reduce the thickness of the selective layer and to improve mechanical stability [26]. Chrzanowska et al. [26] prepared the PA6 supported chitosan nanocomposite membranes for the dehydration of ethanol and isopropanol. It was found that the supported membranes possess significantly higher transport properties in comparison with the chitosan membranes. Moreover, it was proven that the preparation of the PA6 supported membranes increased mechanical by increasing the elongation at break [26]. 

The aim of this work was to design and characterize novel membranes for organic–organic pervaporation. PVA based heterogeneous membranes containing the nanofillers (NaY zeolite) were fabricated. The prepared membranes were applied in the pervaporation of organic–organic mixture (i.e., ethanol/ethyl *tert*-butyl ether). Additionally, the impact of the presence of water traces in feed solution on the separation efficiency was discussed.

## 2. Materials and Methods 

### 2.1. Reagents and Solvents

Ethanol (pure 99.8%), formic and acetic acids were acquired from Chempur (Piekary Śląskie, Poland). Ethyl *tert*-butyl ether was kindly provided by PKN Orlen S.A. (Płock, Poland). 

Poly (vinyl alcohol) (PVA) powder (Elvanol 71-30, fully hydrolyzed, the molecular weight of 100 kDa) was kindly delivered by Kuraray Co. Ltd. (Chiyoda, Tokyo, Japan). NaY zeolite and glutaraldehyde (GA) (25% aqueous solution) were supplied by Abcr GmbH (Karlsruhe, Germany). Poly(amide 6) (PA6) pellets were provided by the ZWCH STILON S.A. (Gorzów Wielkopolski, Poland). Ultrapure reverse osmosis water was used in this study.

### 2.2. Preparation of Composite Membranes

Poly(amide-6) porous supports were prepared following the procedure proposed by Ceynowa and Adamczak [27]. PA6 pellets were dissolved in mixtures comprising of formic acid, acetic acid, calcium chloride, and water (52.4:8.3:8.3:17.5 wt %) to obtain the mixture containing the 13.5 wt % of the polymer. In the next step, the polymer solution was cast on a glass plate using the automatic film applicator (Erichsen Gmbh Co. Kb, Hemer, Germany). The slit of the casting knife and casting speed were equal to 0.4 mm and 10 mm sec^−1^, respectively. The cast polymer solution was left on air for 10 min and subsequently immersed in the water coagulation bath at room temperature. The PA6 supports were finally dried at 25 °C for 24 h.

10 wt % PVA solution was prepared by dissolving the PVA powder in the reverse osmosis water by stirring under the reflux at 100 °C for 6 h. After cooling down the PVA aqueous solution, a proper amount of glutaraldehyde (5 wt % relative to the polymer content in the solution) was added dropwise, and then the PVA/GA solution was stirred for 24 h. 

In the case of the composite membrane with a selective layer containing NaY zeolite, the given amount of NaY powder (corresponding to 10 wt % of the polymer) was added to the PVA/GA solution and stirred for additional 24 h. 

Composite membranes were prepared by casting the PVA/GA or PVA/GA/NaY solution on porous PA6 support using the automatic film applicator. The slit of the casting knife and casting speed were equal to 0.4 mm and 10 mm s^−1^, respectively. Subsequently, the resulting composite membranes were left to dry for 24 h at 25 °C. 

### 2.3. Material Characterization of NaY and Composite Membranes

Morphology of NaY zeolite and membranes (surface and cross-section) was recorded using LEO 1430 VP microscope (Leo Electron Microscopy Ltd., Cambridge, UK). Before the analysis of the cross-section, the membrane was immersed in liquid nitrogen and broken. Membranes were additionally sputtered with a conductive layer of Au/Pd (the composition of layer 80/20, thickness ca. 5 nm).

TEM analysis of NaY zeolite was performed using Tecnai F20 X-Twin (FEI Europe B.V., Eindhoven, The Netherlands). Suspension of NaY in ethanol was placed on the copper mesh and positioned in the holder.

The surface topography of supported PA6 and composite membranes was accomplished with NanoScope MultiMode SPM system (Veeco Digital Instrument, Plainview, USA) in a tapping mode. The values of roughness parameter (*R_A_*) were determined for the scanned area equal to 10 μm × 10 μm using the Nanoscope v6.13 software (Bruker Optik GmbH, Ettlingen, Germany). Silicon nitride (Si_3_N_4_) probe (NP-1) was used during the measurements. Spring constant value, as provided by the manufacturer (Veeco), was equal to 0.58 Nm^−1^. Ambient temperature conditions were kept during all experiments.

The thermal properties of NaY zeolite and fabricated membranes were studied using the TGA-DTA Thermal Analysis TA Instruments type SDT 2960 (TA Instrument, Champaign, IL, USA). The analysis was carried out at a temperature range from 30 to 900 °C (heating rate 10 °C/min) under nitrogen condition. TA Universal Analysis v5.5.24 software (TA Instrument, Champaign, IL, USA) was implemented for the analysis of obtained thermograms. 

The XRD patterns of NaY were collected in the range of 5°−50° with the scanning speed equal to 0.05°/min using the Philips X”Pert X’ with the Celerator Scientific detector and Cu anode (Malvern Panalytical, Malvern, UK). The diffraction profiles were corrected by linear plotting of background and then by the smoothing cycles. The contribution of Kα2 was eliminated by the Raschinger method. 

Nitrogen adsorption/desorption analysis was recorded using Gemini VI (Micromeritics Instrument Corp., Norcross, USA) at −195.85 °C. Before the analysis, the powder sample of NaY zeolite was degassed at 110 °C for 6 h. BET (Brunauer–Emmett–Teller) and BJH (Barrett–Joyner–Halenda) methods were implemented for calculation the specific surface area and pore size, respectively. 

The particle size distribution of NaY zeolite was determined using the Litsizer™500 (Anthon Paar, Graz, Austria) using the DLS technique. The Kalliope™ v2.10.5 software (Anthon Paar, Graz, Austria) was used for the analysis of the obtained results. Prior to the analysis, the samples were dispersed with ultrasounds for 5 min and then directly diluted to final concentration (100 μg/mL) and analyzed. DLS measurements were performed at 25 °C in deionized water. 

Bruker Vertex 70 (Bruker Optick GmbH, Ettlingen, Germany) spectrometer was used for the recording the FTIR spectra of NaY zeolite power, crosslinked PVA membrane, and PVA-NaY/PA6 composite membrane. Spectra were collected in ATR mode with the German crystal in the range of 400–4000 cm^-1^, resolution equal to 4 cm^−1^, and 1024 scans. Obtained spectra were analyzed using the OPUS 7.5 software (Bruker Optick GmbH, Ettlingen, Germany). 

During the goniometric measurements, the following testing liquids were used, water (72.5 mN m^−1^) and α-bromonaphthalene (44.4 mN m^−1^). The selection of the testing liquids accomplished the requirements of Owens, Wendt, Rabel, and Kaelble method. Contact angle measurements were assisted with the simultaneous analysis of surface topography using Theta Flex Tensiometer (Biolin Scientific, Gothenburg, Sweden) at room temperature. Attention Theta (OneAttension Version 4.02) software was used for data acquisition and processing. The topography module is operating according to the fringe projection phase-shifting method. The advantage of that technique is the possibility of determining the contact angle value corrected by the roughness. During the analysis first, the roughness of the sample is measured and then on the same selected area, the drop of the testing liquid is deposited. 

Pore size and pore size distribution were determined to base on the modified bubble point method [28,29,30] applying Coulter Porometer II from Coulter Electronics Ltd. (Luton, UK). Before each test, the membrane samples (2.5 cm diameter) were immersed in Porofil wetting liquid with surface tension *γ_L_* = 16 mN m^−1^. Measurements were done in triplicate and an average value of pore size was shown.

### 2.4. Pervaporation Experiments

Pervaporation experiments were accomplished at 30 °C using a standard laboratory set up described in detail in our previous work [31]. The membrane specific surface area and temperature of experiments were equal to 14.5 cm^2^ and 30 °C, respectively. Ethanol/ethyl *tert*-butyl ether mixtures were used as feed solutions in the following mass ratios: 15/85, 30/70, 50/50, 60/40, and 75/25 (EtOH/ETBE). 

Transport properties of composite membranes were estimated using the thickness normalized total flux (*J_N,t_*) and thickness normalized partial permeate flux of component *i* (*J_N,i_*).

(1) $$J_{N,t}=l\cdot \frac{\Delta m}{A\cdot \Delta t}\;[\mu m\;g\;m^{-2}\;h^{-1}]$$

(2) $$J_{N,i}=J_{N,t}\cdot y_{i}\;\left[\mu m\cdot g\cdot m^{-2}h^{-1}\right]$$

where *l*—thickness of the dense selective layer [μm], *Δm*—a mass of permeate sample [g], *Δt* time of collecting the permeate sample [h], *A*—membrane active area [m^2^], *y_i_*—a mass fraction of component *i* in permeate [-].

Separation factor (β)–Equation (3) and thickness normalised Pervaporation Separation Index (PSI_N_)–Equation (4) were implemented for the evaluation of the separation properties of the composite membranes.

(3) $$\beta =\frac{y_{i}/\left(1-y_{i}\right)}{x_{i}/\left(1-x_{i}\right)}\;[-]$$

(4) $$PSI_{N}=J_{N,t}\cdot \left(\beta -1\right)\;[\mu m\;g\;m^{-2}\;h^{-1}]$$

where *y_i_*—a mass fractions of component *i* in the permeate [-], *x_i_*—a mass fraction of component *i* in the feed [-].

### 2.5. Gas Chromatography

Varian 3300 (Varian, Palo Alto, CA, USA) gas chromatography equipped with thermal conductivity detector (TCD) and Poropack Q column was used for determination of the composition of feed and permeate sample. Borwin v1.21.07 software (JMBS, Grenoble, France) was applied for the analysis of obtained results. 

The values of LOD (limit of detection) and LOQ (limit of quantification) of gas chromatography analysis were also estimated. The procedure of determination of LOD and LOQ is described in detail elsewhere [28]. The determined value of LOD and LOQ are listed in Table 1.

## 3. Results and Discussion

### 3.1. Characterization of NaY Zeolite

Physicochemical properties (crystalline structure, thermal properties, morphology, specific surface area, pore size, and particle size) of commercial NaY zeolite were evaluated using XRD, TEM, SEM, and DLS techniques. 

**Figure 1:**
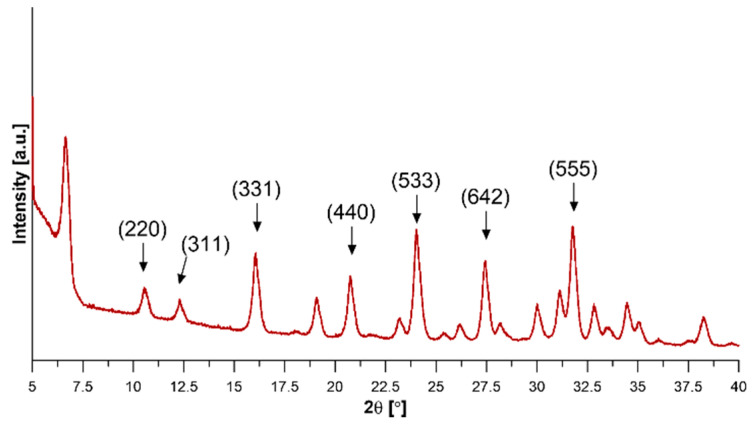
XRD spectra of NaY zeolite.

XRD spectrum of NaY is presented in Figure 1. All diffraction peaks were well-indexed, which means that NaY is characterized by a highly crystalline structure (JCPDs No. 043-0168). Characteristic peaks of NaY zeolite at 2θ equal to 10.3°, 12.1°, 16.1°, 20.7°, 24.0°, 27.4°, and 31.8° correspond to the crystallographic plane (220), (311), (331), (440), (533), (642), and (555), respectively [32].

**Figure 2:**
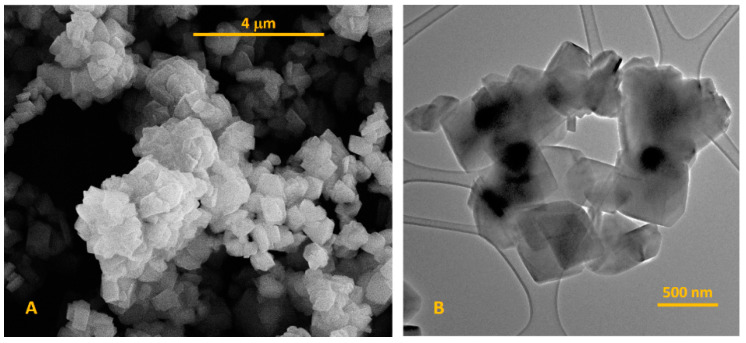
SEM (**A**) and TEM (**B**) micrographs of NaY zeolite.

Scanning electron microscopy and transmission electron microscopy were applied for determination the NaY zeolite particle shape, morphology, and size. Figure 2A presents the SEM micrograph, proving that NaY possesses the octahedron shape of crystals, which are characteristic of this type of compound. The particle size of zeolite was determined from the TEM analysis (Figure 2B). Particle size was evaluated using the ImageJ v 1.80_112 (NIH, Bethesda, MD, USA). The determined particle size of NaY zeolite was in the range of 520–690 nm. Moreover, it was also observed that the NaY zeolite particles show a tendency to form agglomerates.

**Figure 3:**
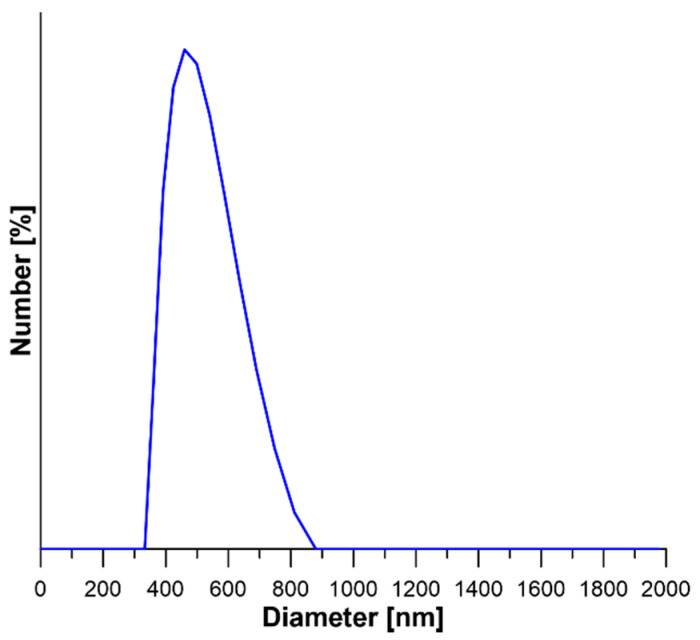
DSL of NaY particle size distribution.

The particles size distribution of NaY zeolite was assessed with Dynamic Light Scattering (DLS)—Figure 3. The particle size distribution of commercial NaY zeolite was in the range of 360–811 nm, with the statistical average particle size equal to 498 nm. The value of particle sizes obtained from the DLS analysis demonstrates compliance with the results obtained from the TEM analysis. It is worth noting that the value of the particle size of this type of zeolite depends on the synthesis procedure. Mu et al. [33] applied two different synthesis procedures of NaY with seeding prepared with sodium silicon and silica sol. In the case of the former synthesis route, the particle size distribution was in the range of 60–155 nm, while for the NaY synthesized for the seeding with silica sol, this value was in the range 670–910 nm [33]. 

**Figure 4:**
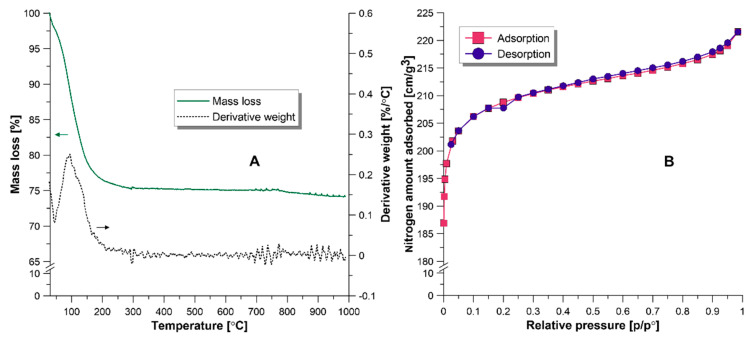
(**A**)—TGA and DTG curves of NaY zeolite. (**B**)—N_2_ adsorption isotherm of NaY.

Figure 4A presents the results of the thermogravimetric analysis. The mass change in the range of 50–200 °C corresponds to the loss of adsorbed water [34,35]. Further increase in temperature does not cause any significant mass loss of zeolite sample. The observed weight loss indicated the final decomposition products of NaY zeolite are SiO_4_ and AlO_4_ [34,35]. N_2_ adsorption/desorption isotherm was performed to evaluate the specific surface area, pore size, and pore volume of zeolite (Figure 4B).

Obtained adsorption curve showed a sharp increase of adsorbed N_2_ in the low relative pressure regions. This type of isotherm is characteristic of the microporous materials (isotherm type II, according to the IUPAC) [36]. Nitrogen adsorption/desorption isotherm was also used for the determination of the BET surface area, pore size, and BJH pore volume. Obtained results and comparison with literature data are displayed in Table 2. Mu et al. [33] observed that the particles size influences the specific surface area and pore volume. NaY-100 possesses a smaller crystal (100 nm) comparing with NaY-500 (500 nm). Obtained results showed that the zeolite with smaller particle size is characterized by a higher BET value and pore volume [33]. Moreover, the commercial NaY zeolite, which has similar particle size to NaY-500, exhibits a similar specific surface area and pore volume.

### 3.2. Characteristics of Composite Membranes

Physiochemical properties morphology, thermal stability, and surface properties of composite membranes were evaluated using SEM, AFM, TGA, and goniometry techniques. 

**Figure 5:**
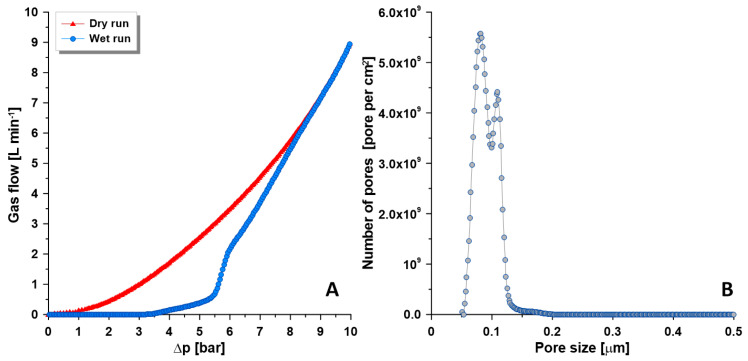
Porosity of PVA porous support. (**A**) gas fluxes in wet and dry runs; (**B**) pore size distribution.

The composite PVA/PA6 and PVA-NaY/PA6 membranes consist of the porous PA6 support and thin dense selective layer. The pore size of porous PA6 support was determined using a modified bubble point method [28,29,30]. Figure 5 presents the pore size distributions of the porous support. The pore size of porous support was calculated based on ‘wet’ and ‘dry’ runs of apparatus. During the ‘wet run’ the measuring gas removes the liquid (Porofil) from the pores, while during the ‘dry run’ the measuring gas flows through the dry membrane (Figure 5A). The measurement starts from the biggest pores and gas pressure continuously increase during the measurement [30]. The pore size, as well as pore size distribution, has a direct and significant influence on the transport features (Figure 5B). In the composite membranes dedicated to pervaporation, it is required to generate porous support and a dense selective layer. The average pore size of the prepared PA6 support membrane was equal to 0.107 μm. The pore size distribution possesses a narrow peak in the range of 0.073 and 0.122 μm (Figure 5B).

**Figure 6:**
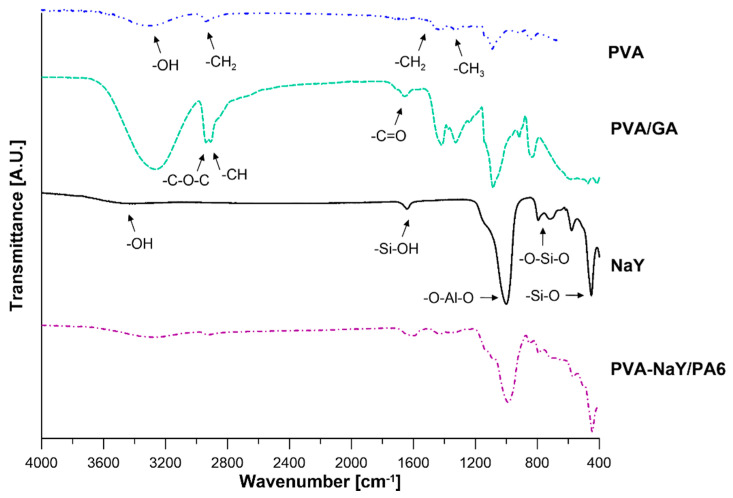
FTIR spectra of PVA pristine membrane, PVA membrane crosslinked with glutaraldehyde (PVA/GA), zeolite (NaY) filler, and the composite membrane (PVA-NaY/PA6).

FTIR analysis was performed to confirm the crosslinking reaction of PVA membranes. The results are presented in Figure 6. The strong band at around 3400 cm^−1^ corresponds to the stretching vibration of –OH group of PVA. Moreover, the bands at 1414 cm^−1^ and 1327 cm^−1^ refer to the –CH_2_ and –CH_3_ bending vibration of the PVA matrix [37]. PVA crosslinking reaction occurs through the formation of the acetal bridges and reduces the number of free –OH groups [38]. As a result of the crosslinking reaction, two new peaks appeared in the spectrum. The peaks at 2940 cm^−1^ and 2908 cm^−1^ correspond to the stretching vibrations of –C–O–C and –CH of aldehyde group [38] (Figure 6). The additional peak at 1657 cm^−1^ refers to the –C=O group of glutaraldehyde [38] (Figure 6).

In the case of NaY zeolite, several characteristic peaks can be observed (Figure 6). Peaks located at 3400 cm^−1^, 1643 cm^−1^, 1000 cm^−1^, 717 cm^−1^, 580 cm^−1^, and 450 cm^−1^ are related to the OH stretching vibrations from Si–OH, Si–OH bending vibration, O–Al–O asymmetry stretch vibration, stretching vibration Si–O of O–Si–O bonds, stretching vibration Si–O of Si–O–Si bonds, and Si–O bending vibration of Si–O–Si bonds, respectively [37] (Figure 6).

Taking into account the spectrum of the selective layer of PVA-NaY/PA6 membrane, characteristic peaks from the crosslinked PVA and NaY zeolite were also found (Figure 6) related to the stretching vibration of –OH group from PVA matrix and NaY zeolite, vibrations of –CH, –C–O–C, and –C=O from glutaraldehyde, and vibrations of –O–Al–O, –Si–O, –SiOH groups, from NaY zeolite.

**Figure 7:**
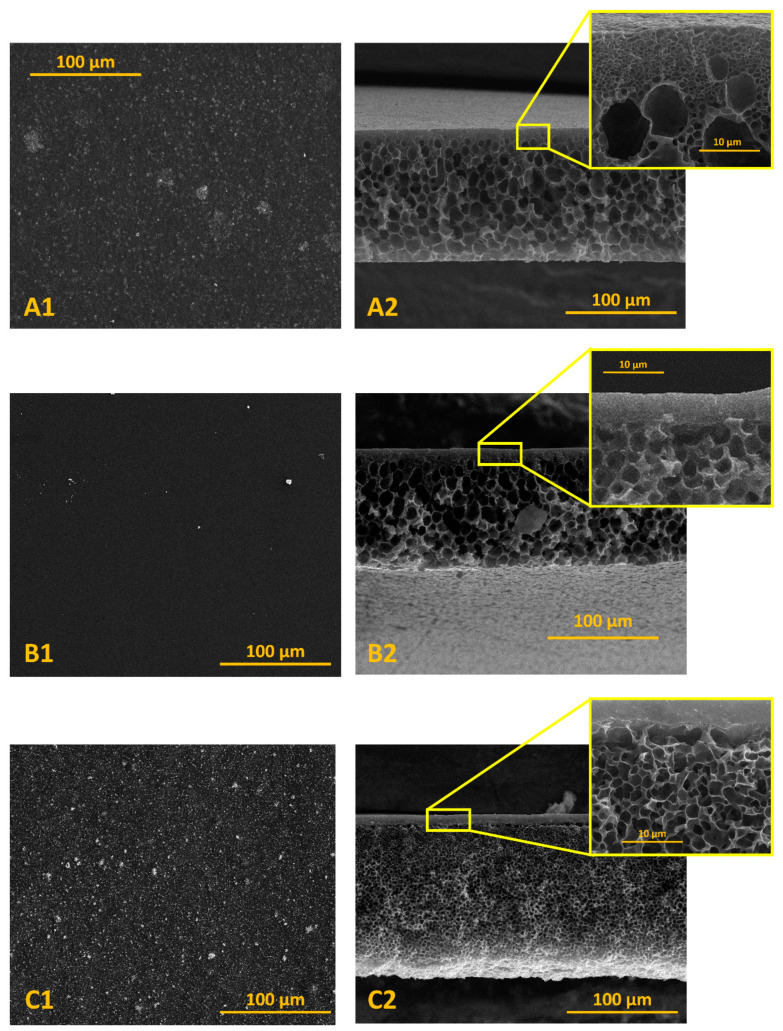
SEM micrographs of surface and cross-section of porous support PA6 (**A1** and **A2**), PVA/PA6 (**B1** and **B2**), and PVA-NaY/PA6 (**C1** and **C2**) composite membranes.

Morphology of porous support and composite membranes were evaluated using the SEM analysis. In the case of PA6 support, SEM surface micrographs revealed the visible pores on the surface (Figure 7(A1)). Analysis of surface morphology of PVA/PA6 and PVA-NaY/PA6 composite membranes showed that the surfaces of these membranes are dense without pores and uniform (Figure 7(B1,C1)). It can be concluded that the NaY zeolite particles are well dispersed in the PVA matrix (agglomerates were not visible). 

Figure 7(A1,B1,C1) present the cross-section micrographs of porous PA6 support and composite membranes. Analysis of Figure 7A proved the formation of porous PA6 membranes with homogeneous pore distribution. In the case of composite membranes (Figure 7(A1,C1)), SEM of the cross-sections confirmed that composite membranes consist of porous PA6 support and a dense selective top layer. Moreover, it can be also noticed that NaY particles are distributed in the PVA matrix. The thickness of the selective layer was evaluated using ImageJ v 1.80_112 (NIH, Bethesda, MD, USA). The thickness of porous support was in the range of 128–130 μm whereas the thickness of the selective layer was in the range of 3.7–4.3 μm.

**Figure 8:**
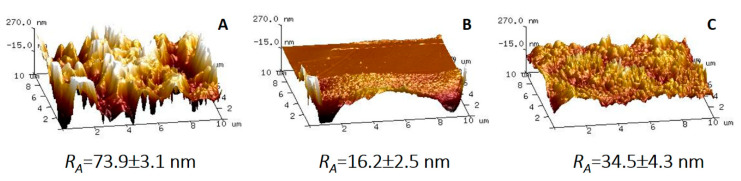
Surface topography and average values of *R_A_* of porous PA6 support (**A**), PVA/PA6 membrane (**B**), and PVA-NaY/PA6 membrane (**C**).

The topography of the porous support and composite membranes was analyzed using atomic force microscopy and discussed basing on the average roughness parameter (*R_A_*) [39]. The obtained results are presented in Figure 8. 

Figure 8 shows the differences in the surface topography of the PA6 support and composite membranes. According to the presented data, the highest value of *R_A_* parameters was found for the PA6 support, which is related to the porous structure of this membrane (Figure 8A). The presence of pores on the surface of the PA6 membrane causes the high surface roughness. It should be also mentioned that these results are in a very good accordance with the SEM analysis (Figure 7(A1,A2)). In the case of PVA/PA6 membrane, it was found that the surface of this composite membrane is smoother than the surface of PA6 membrane, and it is characterized by a lower roughness parameter (*R_A_* = 16.6 nm). It can be concluded that the application of a thin layer on a porous support leads to the creation of the uniform surface. These differences were noticed in the AFM phase image (Figure S1), where two types of polymeric phases were detected. PVA support layer possessed porous structure that was observed on the SEM as well as on the phase AMF. Moreover, the selective layer was characterized by a smooth and compact structure. Obtained results showed also that introduction of NaY zeolite to the PVA matrix influences the surface roughness. As a result of membrane modification, the value of *R_A_* increases from 16.6 to 34.5 nm (Figure 8C). A similar observation was reported by Chrzanowska et al. [26]. Authors noticed that PA6/Ch and PA6/Ch/MMT composite membranes possess lower surface roughness (7.2 and 10.8 nm) compared with porous PA6 support (63.1 nm) [26].

**Figure 9:**
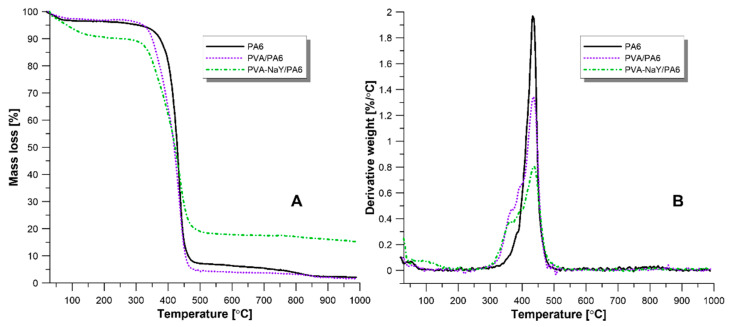
TGA (**A**) and DTG (**B**) curves of PA6 support, PVA/PA6 and PVA-NaY/PA6 composite membranes.

Thermal properties of the porous support and composite membranes were determined using thermogravimetric analysis. The thermal degradation curves are presented in Figure 9. The degradation of porous PA6 support occurs in one step, which corresponds to the decomposition of the polyamide network. The porous PA6 is thermally stable up to 400 °C (Figure 9A).

According to the literature, the thermal degradation of pristine PVA is a process presiding in three steps [37]. First mass loss (around 100 °C) corresponds to the loss of adsorbed water molecules. The second stage is related to the loss of –OH groups and the deacetylation of PVA chains (200–380 °C) while the third stage corresponds to the degradation of the PVA backbone (400–500 °C) [37]. Obtained results showed that in the case of crosslinked PVA/PA6 composite membrane is thermally stable up to 350 °C. During the crosslinking reaction, glutaraldehyde reacts with the hydroxyl group of PVA and creates the acetal bridges [38]. After crosslinking reaction, the number of –OH groups on the surface decreased which increasing the thermal stability of crosslinked PVA. A similar trend was found by Gebru and Das [40]. The authors observed that crosslinking of PVA leads to the increasing thermal stability from 250 to 300 °C [40]. It should be also mentioned that the third stage corresponding to the degradation of PVA chains overlaps with the degradation peak of PA6 support (Figure 9A,B). 

In the case of PVA-NaY/PA6 composite membrane, the first mass loss is related to the removal of entrapped water in the NaY zeolite and in the PVA matrix (Figure 9A,B). Similarly, to the PVA/PA6 composite membrane, the next degradation stage starts at 350 °C and it is a combination of decomposition of both PA6 and PVA matrices.

**Figure 10:**
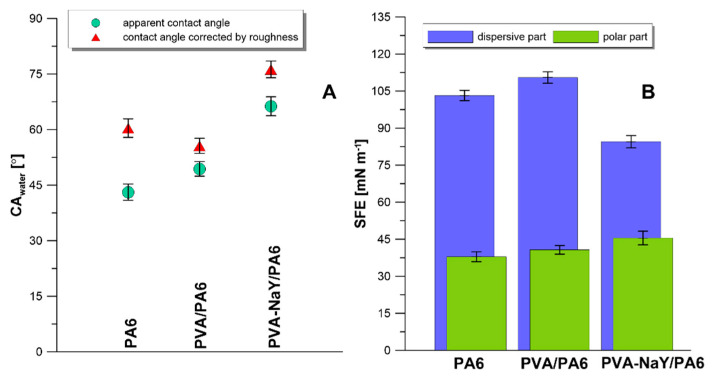
Apparent and corrected contact angle (CA) of water (**A**) and surface free energy (**B**) for porous support, PVA/PA6, and PVA/NaY/PVA composite membranes.

In the characterization of the new material, it is essential to define its wettability behavior. In the presented research, the goniometric measurements were coupled with direct analysis of surface topography. By implementing the fringe projection phase-shifting method, it was possible to measure the apparent contact angle and contact angle corrected by the roughness [41,42]. The data are presented in Figure 10A and Figure S2. The corrected contact angle of water for the investigated samples was equal to 60.4°, 55.6°, and 75.2° for PA6, PVA/PA6, and PVA-NaY/PA6, respectively (Figure 10A). These values were varied from the apparent data, which were following 43.1°, 49.4°, and 66.3° for PA6, PVA/PA6, and PVA-NaY/PA6, respectively. The differences are related to the impact of the roughness of the surface as well as the introduction of the new components. The biggest difference between corrected and apparent contact angle was observed for PA6 sample which is consistent with the roughness presented in Figure 8 and data presented in Figure S2. Owing to the very low level of heterogeneity of the PVA/PA6 sample, the difference between apparent and corrected by roughness CA is only ca. 6°. However, for the sample with the zeolite filler, the difference between the mentioned parameters was equal to almost 9°, and it was directly related to the introduction of a hydrophilic additive (Figure 10A). Although the topography is characterized by an optical method, the differences in a big scale of magnification were also detected. Particularly, the presence of NaY in the selective layer of the membrane causes the increases of surface heterogeneity. A similar relationship was found by Kim et al. [43]. Authors observed that casting a thin layer of PVA on PA6 membrane caused the decrease of the value of *CA_water_* from ca. 43° to ca. 38° [43]. The registered changes in the SFE values also supported the efficient modification of the membranes. An increase of SFE for the PVA/PA6 sample was related to the fact that PVA possesses a more hydrophilic character comparing with PA6 (Figure 10B). However, the reduction of overall SFE and the simultaneous rise in its polar component are associated with the presence of hydrophilic NaY structure.

### 3.3. Pervaporation Experiments

The efficiency of prepared PVA/PA6 and PVA-NaY/PA6 composite membranes were evaluated in pervaporative separation of ethanol/ethyl *tert*-butyl ether containing 15–75 wt % of ethanol. The thickness of a selective layer of PVA/PA6 and PVA-NaY/PA6 composite membranes determined from the SEM micrographs (Figure 7(B2,C2)) was equal to 3.7 and 4.2 μm, respectively.

**Figure 11:**
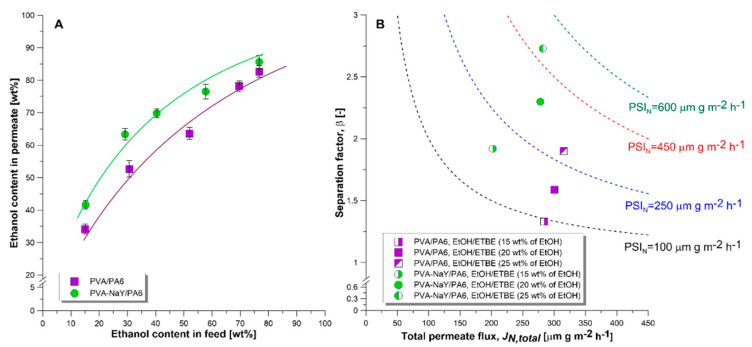
(**A**) McCabe–Thiele separation diagram for investigated membranes in contact with ETBE/EtOH mixture. (**B**) Comparison of membrane’s efficiency of PVA/PA6 and PVA-NaY/PA6 membranes in the removal of ethanol from ETBE/EtOH mixture containing 15, 20, and 25 wt % of EtOH. Dashed lines correspond to the following values of PSI_N_: black – PSI_N_ = 100 μm g m^−2^ h^−1^, blue – PSI_N_ = 250 μm g m^−2^ h^−1^, red – PSI_N_ = 450 μm g m^−2^ h^−1^, and green – PSI_N_ = 600 μm g m^−2^ h^−1^.

Figure 11A presents the McCabe–Thiele separation diagram acquired during the separation of EtOH/ETBE mixture containing the 15–75 wt % of EtOH. It was established that tested composite membranes selectively transport ethanol from the feed solution. Moreover, the ethanol content in permeate increases with increasing ethanol content in the feed mixture. Preferential ethanol transport from the EtOH/ETBE mixture results from the polar character of both PVA matrix and NaY zeolite. Comparing the polarity of the separated components, ethanol is characterized by the higher polarity comparing with the ETBE [44].

**Figure 12:**
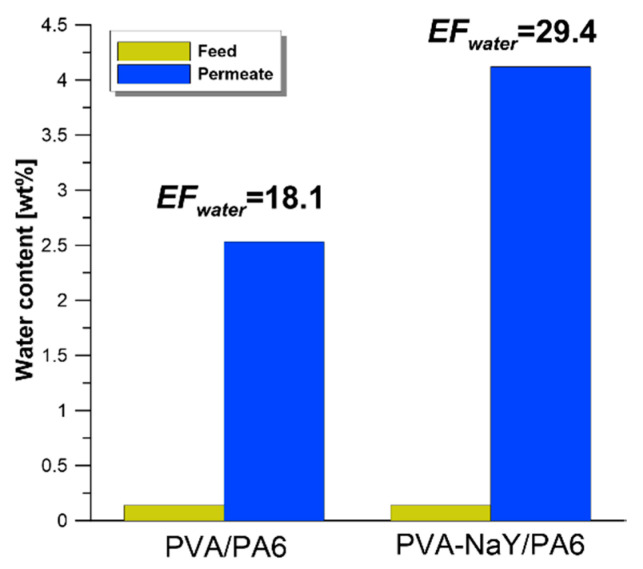
Comparison of enrichment factor of water obtained during the separation of EtOH/ETBE mixture containing 20 wt % of EtOH.

Pervaporation separation index (*PSI_N_*) is a commonly used parameter for the assessment of the efficiency of various membranes applied for the separation of the same mixture. Figure 11B presents a comparison of the efficiency of PVA/PA6 and PVA-NaY/PA6 membranes in the separation of EtOH/ETBE mixtures containing 15, 20, and 25 wt % of EtOH. As it can be seen from Figure 12 the PVA-NaY composite membrane showed higher separation (higher value of *PSI_N_* and *β*) ability for removal of EtOH from the EtOH/ETBE mixture comparing with the PVA/PA6 one. The values of *β* (2.3) and *PSI_N_* (288 μm g m^−2^ h^−1^) for PVA-NaY/PA6 composite membrane were 143% and 160% higher than those for the PVA/PA6 composite membrane (*β* = 1.6, *PSI_N_* = 180 μm g m^−2^ h^−1^) during the separation of the azeotropic mixture (20 wt % of EtOH). NaY zeolite possesses the pore size larger (10.1 Å, Table 2) than the kinetic diameter of ethanol (4.5 Å [45]) and ethyl *tert*-butyl ether, therefore EtOH and ETBE molecules can pass through the pores of NaY zeolite. However, due to the polar character of the NaY, this zeolite attracts ethanol molecules more preferably than less polar ethyl *tert*-butyl ether, which leads to the increase of membrane selectivity for ethanol. 

It should be also noticed that the reduction of total permeate flux was observed with an introduction of NaY into a PVA polymer matrix (Figure 11B). This observation could be related to the increased stiffness of the PVA matrix resulting from the incorporation of NaY zeolite [46]. A similar relationship was found by Kurşun [47] during the separation of water/isopropanol mixture containing 40 wt % of water. Pervaporation results showed that an increase in NaY content from 0.1 to 0.5 wt % in PVA matrix caused a decrease in total permeate flux from ca. 0.33 kg m^−2^ h^−1^ to ca. 0.28 kg m^−2^ h^−1^ with a simultaneous moderate increase in selectivity from *β_water_* = 23.5 to *β_water_* = 24.4 [47].

### 3.4. Influence of Water Presence on Separation Effectiveness

Water presence in organic solvents is a crucial issue at the industrial scale. High water content in permeate can contribute to phase separation or can lead to corrosion of the piping and storage tanks set up. 

In our previous works [48,49], the enrichment factor for water (*EF_water_*) was applied for the assessment of water influence on the resultant efficiency of the pervaporation separation process (Equation (5)) [48,49]. Value of enrichment factor higher than 1 indicates that water is transported preferentially from feed to permeate.

(5) $$EF_{water}=\frac{P_{w}}{F_{w}}\;[-]$$

where *P_w_*—water content in permeate (wt %), *F_w_*—water content in feed (wt %).

Water content in the feed solution was in the range of 0.14–0.41 wt %. As it can be seen from Figure 12 both PVA/PA6 and PVA-NaY/PA6 composite membranes showed a value of *EF_water_* higher than one. It was noticed that water content in permeate increased from 0.14 wt % (water content in feed) to 2.53 and 4.12 wt % for PVA/PA6 and PVA-NaY/PA6 composite membranes, respectively. According to the obtained *CA_water_*, both PVA/PA6 and PVA-NaY/PA6 composite membranes possess hydrophilic character. It should be also highlighted that in contact with organic solvents, polymeric membranes swell. The transport of separated components in swollen membranes is facilitated. Moreover, water has a lower kinetic diameter (2.65 Å [50]) comparing with ethanol (4.5 Å [45]) and is much more polar than ethanol [44]. Due to the lower kinetic diameter and higher polar properties, water molecules can be easily transported through the swollen, hydrophilic PVA based membranes.

Organic solvents are widely used in a variety of industrial sectors. Reclaiming and recycling the solvents may be the most environmentally and economically beneficial option for managing consumed solvents. To meet reuse specifications of the purifying solvents can be really challenging. Particularly for hydrophilic solvents, water must be removed prior to reuse, yet many hydrophilic solvents form hard-to-separate azeotrope mixtures with water. Unfortunately, such mixtures make separation processes energy-intensive, creating further economic challenges. 

Higher *EF_water_* for PVA-NaY/PA6 membrane is related to the higher level of hydrophilicity (higher value of polar part of SFE–Figure 10B) of this membrane compared with PVA/PA6 one. It can be stated that hydrophilic membranes can be also applied for the removal of excess water from the feed. Obtained results are consistent with our recently published works [48,49].

## 4. Conclusions

A novel type of the polymeric PVA based membranes supported on PA6 with the inorganic zeolite filler (NaY) possessing enhanced separation properties was fabricated. The addition of NaY improved membrane performance in the pervaporation process. Generally, stable and efficient membrane materials were obtained. Composite materials were implemented for the separation of industrially valuable solvents ethanol and ethyl *tert*-butyl. As an effect of NaY introduction, the development of membrane efficiency was observed. The values of separation factor *β* and *PSI_N_* for filled PVA-NaY/PA6 membrane were improved by 143% and 160%, referring to pristine PVA/PA6 one. Ethanol was selectively transported across the membrane from the EtOH/ETBE mixture. The relation between apparent and corrected contact angle of water was determined and discussed. The biggest differences were noticed for the highly rough surface, i.e., porous PA6. An impact of water traces on the separation features has been also studied. The level of water in the feed was equal to 0.14 wt %, and after the separation process, the water levels in permeates were equal to 2.53 and 4.12 wt % for PVA/PA6 and PVA-NaY/PA6 composite membranes, respectively. 

## Figures and Tables

**Table 1:** LOD and LOQ for investigated organic solvents.

Solvent	LOQ (%)	LOD (%)
ETBE	0.16	0.20
EtOH	0.03	0.09
H_2_O	0.03	0.11

**Table 2:** Comparison of BET surface area, pore size, and pore volume of NaY.

	BET (m^2^ g^−1^)	Pore Size (Å)	Pore Volume (cm^3^ g^−1^)	Ref.
NaY-586	666	10.1	0.32	this work
NaY-100	728	20.4	0.41	[33]
NaY-500	671	20.3	0.38	[33]

## Supplementary Materials

The following are available online at https://www.mdpi.com/1996-1944/13/17/3676/s1.

## References

[1] Touchal S., Roizard D., Perrin L. (2006). Pervaporation properties of polypyrrolidinone-based membranes for EtOH/ETBE mixtures separation. J. Appl. Polym. Sci..

[2] Williams P.R.D. (2001). MTBE in California Drinking Water: An Analysis of Patterns and Trends. Environ. Forensics.

[3] Galán G., Martín M., Grossmann I. (2019). Integrated Renewable Production of ETBE from Switchgrass. ACS Sustain. Chem. Eng..

[4] Yee K.F., Mohamed A.R., Tan S.H. (2013). A review on the evolution of ethyl tert-butyl ether (ETBE) and its future prospects. Renew. Sustain. Energy Rev..

[5] Nicholls H.C.G., Mallinson H.E.H., Rolfe S.A., Hjort M., Spence M.J., Thornton S.F. (2020). Influence of contaminant exposure on the development of aerobic ETBE biodegradation potential in microbial communities from a gasoline-impacted aquifer. J. Hazard. Mater..

[6] de Menezes E.W., Cataluña R. (2008). Optimization of the ETBE (ethyl tert-butyl ether) production process. Fuel Process. Technol..

[7] Hassan Hassan Abdellatif F., Babin J., Arnal-Herault C., Nouvel C., Six J.-L., Jonquieres A. (2017). Bio-based membranes for ethyl tert-butyl ether (ETBE) bio-fuel purification by pervaporation. J. Membr. Sci..

[8] Volkov V.V. (1994). Separation of liquids by pervaporation through polymeric membranes. Russ. Chem. Bull..

[9] Luis P., Luis P. (2018). Chapter 3—Pervaporation. Fundamental Modelling of Membrane Systems.

[10] Baker R. (2004). Membrane Technology and Applications.

[11] Číhal P., Vopička O., Lanč M., Kludský M., Velas J., Hrdlička Z., Michalcová A., Dendisová M., Friess K. (2018). Poly(butylene succinate)-cellulose triacetate blends: Permeation, pervaporation, sorption and physical structure. Polym. Test..

[12] Dutta B.K., Sikdar S.K. (1991). Separation of azeotropic organic liquid mixtures by pervaporation. Aiche J..

[13] Olsson J., Trägårdh G., Lipnizki F. (2002). The influence of permeant and membrane properties on mass transfer in pervaporation of volatile organic compounds from dilute aqueous solutions. Sep. Sci. Technol..

[14] Wang M., Arnal-Herault C., Rousseau C., Palenzuela A., Babin J., David L., Jonquieres A. (2014). Grafting of multi-block copolymers: A new strategy for improving membrane separation performance for ethyl tert-butyl (ETBE) bio-fuel purification by pervaporation. J. Membr. Sci..

[15] Hassan Hassan Abdellatif F., Babin J., Arnal-Herault C., David L., Jonquieres A. (2016). Grafting of cellulose acetate with ionic liquids for biofuel purification by a membrane process: Influence of the cation. Carbohydr. Polym..

[16] Zereshki S., Figoli A., Madaeni S.S., Galiano F., Drioli E. (2011). Pervaporation separation of ethanol/ETBE mixture using poly(lactic acid)/poly(vinyl pyrrolidone) blend membranes. J. Membr. Sci..

[17] Jonquières A., Clément R., Lochon P. (2005). New film-forming poly(urethane-amide-imide) block copolymers: Influence of soft block on membrane properties for the purification of a fuel octane enhancer by pervaporation. Eur. Polym. J..

[18] Assabumrungrat S., Kiatkittipong W., Praserthdam P., Goto S. (2003). Simulation of pervaporation membrane reactors for liquid phase synthesis of ethyl tert-butyl ether from tert-butyl alcohol and ethanol. Catal. Today.

[19] Zhu M., Huang S., Gong Y., Zhou Y., Chen X., Liu Y., Hu N., Zhang F., Chen X., Kita H. (2019). Effect of flouride on preparation and pervaporation performance of NaY zeolite membrane for EtOH/ETBE mixture. Microporous Mesoporous Mater..

[20] Ramezani H., Azizi S.N., Hosseini S.R. (2017). NaY zeolite as a platform for preparation of Ag nanoparticles arrays in order to construction of H_2_O_2_ sensor. Sens. Actuators B.

[21] Broach R.W., Kulprathipanja S. (2010). Zeolite Types and Structures. Zeolites in Industrial Separation and Catalysis.

[22] Singleton N.L., Huddersman K.D., Needham M.I. (1998). The adsorption properties of NaY zeolite for separation of aromatic triazoles. J. Chem. Soc. Faraday Trans..

[23] Sawamura K.-i., Furuhata T., Sekine Y., Kikuchi E., Subramanian B., Matsukata M. (2015). Zeolite Membrane for Dehydration of Isopropylalcohol—Water Mixture by Vapor Permeation. ACS Appl. Mater. Interfaces.

[24] Kita H., Fuchida K., Horita T., Asamura H., Okamoto K. (2001). Preparation of Faujasite membranes and their permeation properties. Sep. Purif. Technol..

[25] Rhim J.-W., Kim Y.-K. (2000). Pervaporation separation of MTBE—Methanol mixtures using cross-linked PVA membranes. J. Appl. Polym. Sci..

[26] Chrzanowska E., Gierszewska M., Kujawa J., Raszkowska-Kaczor A., Kujawski W. (2018). Development and Characterization of Polyamide—Supported Chitosan Nanocomposite Membranes for Hydrophilic Pervaporation. Polymers.

[27] Ceynowa J., Adamczak P. (1992). Enzyme membrane based upon polyamide-6 for oil hydrolysis. J. Appl. Polym. Sci..

[28] Kujawski W., Adamczak P., Narebska A. (1989). A Fully Automated System for the Determination of Pore Size Distribution in Microfiltration and Ultrafiltration Membranes. Sep. Sci. Technol..

[29] Hernández A., Calvo J.I., Prádanos P., Tejerina F. (1996). Pore size distributions in microporous membranes. A critical analysis of the bubble point extended method. J. Membr. Sci..

[30] Szczerbińska J., Kujawski W., Arszyńska J.M., Kujawa J. (2017). Assessment of air-gap membrane distillation with hydrophobic porous membranes utilized for damaged paintings humidification. J. Membr. Sci..

[31] Kujawska A., Knozowska K., Kujawa J., Kujawski W. (2016). Influence of downstream pressure on pervaporation properties of PDMS and POMS based membranes. Sep. Purif. Technol..

[32] Sivakumar K., Santhanam A., Natarajan M., Velauthapillai D., Rangasamy B. (2016). Seed-Free Synthesis and Characterization of Zeolite Faujasite Aluminosilicate Coating on α-Alumina Supports. Int. J. Appl. Ceram. Technol..

[33] Mu L., Feng W., Zhang H., Hu X., Cui Q. (2019). Synthesis and catalytic performance of a small crystal NaY zeolite with high SiO_2_/Al_2_O_3_ ratio. RSC Adv..

[34] Ramezani H., Azizi S.N., Cravotto G. (2019). Improved removal of methylene blue on modified hierarchical zeolite Y: Achieved by a “destructive-constructive” method. Green Process. Synth..

[35] Cele M., Friedrich H., Bala M. (2013). A study of Fe (III) TPPCl encapsulated in zeolite NaY and Fe (III) NaY in the oxidation of n-octane, cyclohexane, 1-octene and 4-octene. React. Kinet., Mech. Catal..

[36] Sing K.S.W., Everett D.H., Haul R.A.W., Moscou L., Pierotti R.A., Rouquérol J., Siemieniewska T. (1985). Reporting Physisorption Data for Gas./Solid Systems With Special Reference to the Determination of Surface Area and Porosity.

[37] Rynkowska E., Fatyeyeva K., Marais S., Kujawa J., Kujawski W. (2019). Chemically and Thermally Crosslinked PVA-Based Membranes: Effect on Swelling and Transport Behavior. Polymers.

[38] Alves P.M.A., Carvalho R.A., Moraes I.C.F., Luciano C.G., Bittante A.M.Q.B., Sobral P.J.A. (2011). Development of films based on blends of gelatin and poly(vinyl alcohol) cross linked with glutaraldehyde. Food Hydrocolloids.

[39] Williams P.M., Drioli E., Giorno L. (2015). Membrane Roughness. Encyclopedia of Membranes.

[40] Das C., Gebru K. (2017). Response Surface Optimization of Electro-Spun Polyvinyl Alcohol Nano-Fiber Membrane Process Parameters and its Characterization. J. Membr. Sep. Technol..

[41] Zuo C., Feng S., Huang L., Tao T., Yin W., Chen Q. (2018). Phase shifting algorithms for fringe projection profilometry: A review. Opt. Lasers. Eng..

[42] Kujawski W., Li G., Van der Bruggen B., Pedišius N., Tonkonogij J., Tonkonogovas A., Stankevičius A., Šereika J., Jullok N., Kujawa J. (2020). Preparation and Characterization of Polyphenylsulfone (PPSU) Membranes for Biogas Upgrading. Materials.

[43] Kim H.J., Baek Y., Choi K., Kim D.-G., Kang H., Choi Y.-S., Yoon J., Lee J.-C. (2014). The improvement of antibiofouling properties of a reverse osmosis membrane by oxidized CNTs. RSC Adv..

[44] Reichardt C., Welton T. (2008). Solvents and Solvent Effects in Organic Chemistry.

[45] Sankar Ganesh R., Navaneethan M., Mani G.K., Ponnusamy S., Tsuchiya K., Muthamizhchelvan C., Kawasaki S., Hayakawa Y. (2017). Influence of Al doping on the structural, morphological, optical, and gas sensing properties of ZnO nanorods. J. Alloys Compd..

[46] Mehrasa M., Anarkoli A.O., Rafienia M., Ghasemi N., Davary N., Bonakdar S., Naeimi M., Agheb M., Salamat M.R. (2016). Incorporation of zeolite and silica nanoparticles into electrospun PVA/collagen nanofibrous scaffolds: The influence on the physical, chemical properties and cell behavior. Int. J. Polym. Mater. Polym. Biomater..

[47] Kurşun F. (2020). Application of PVA-b-NaY zeolite mixture membranes in pervaporation method. J. Mol. Struct..

[48] Knozowska K., Kujawski W., Zatorska P., Kujawa J. (2018). Pervaporative Efficiency of Organic Solvents Separation Employing Hydrophilic and Hydrophobic Commercial Polymeric Membranes. J. Membr. Sci..

[49] Knozowska K., Li G., Kujawski W., Kujawa J. (2020). Novel heterogeneous membranes for enhanced separation in organic-organic pervaporation. J. Membr. Sci..

[50] Scholes C., Kentish S., Stevens G. (2008). Carbon Dioxide Separation through Polymeric Membrane Systems for Flue Gas Applications. Recent Pat. Chem. Eng..

