# Supplementary material for: A New Type of Composite Membrane PVA-NaY/PA-6 for Separation of Industrially Valuable Mixture Ethanol/Ethyl Tert-Butyl Ether by Pervaporation
Source: Materials (Basel). 2020 Aug 20;13(17):3676. doi: 10.3390/ma13173676 (PMC7504003; doi:10.3390/ma13173676)
Supplement: Supplementary file 1 [file materials-13-03676-s001.pdf]

materials

Supporting materials

A new type of composite membrane PVA-NaY/PA-6 for separation of industrially valuable mixture ethanol/ethyl *tert*-butyl ether by pervaporation

Katarzyna Knozowska [1], Joanna Kujawa [1], Renars Lagzdins [1,2], Alberto Figoli [3], and Wojciech Kujawski [1,4,] *

[1] Nicolaus Copernicus University in Toruń, Faculty of Chemistry, 7 Gagarina Street, 87-100 Toruń, Poland; wkujawski@umk.pl
[2] Faculty of Nature Science and Mathematics, Daugavpils University, 1 Parādes Street, Daugavpils LV-5401, Latvia
[3] Institute on Membrane Technology, CNR-ITM, Via P. Bucci 17c, 87030 Rende (CS), Italy
[4] National Research Nuclear University MEPhI, 31 Kashirskoe Hwy, Moscow 115409, Russia
* Correspondence: wkujawski@umk.pl; Tel.: +48-56-611-45-17 (W.K.)

Received: date; Accepted: date; Published: date

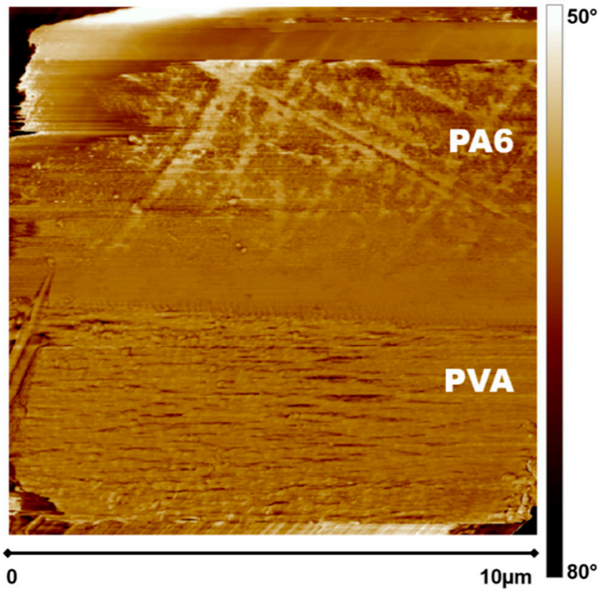

Figure. S1. Phase image for PVA/PA6 membrane.

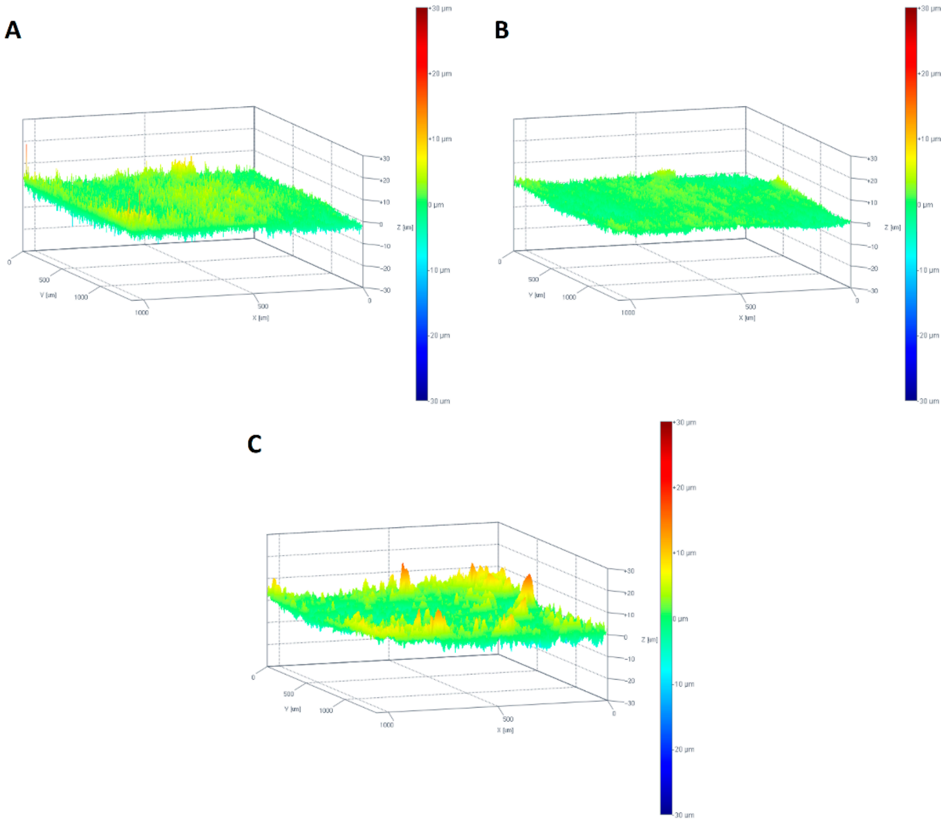

Figure S2. 3-D topography images measured by Theta Flex Tensiometer equipped with 3D Topography module. A - porous support, B - PVA/PA6, C - PVA/NaY/PVA composite membrane.
